# Cystic fibrosis–related kidney disease—emerging morbidity and disease modifier

**DOI:** 10.1007/s00467-025-06715-3

**Published:** 2025-03-17

**Authors:** Merrill Hart, Manish Kumar, Himanshu Ballav Goswami, William Tom Harris, Sladjana Skopelja-Gardner, Agnieszka Swiatecka-Urban

**Affiliations:** 1 University of Virginia, Charlottesville, VA 22903 USA; 2 Department of Pediatrics, University of Alabama at Birmingham, Birmingham, AL 35233 USA; 3 Department of Microbiology and Immunology, Dartmouth Geisel School of Medicine, Lebanon, NH 03756 USA; 4 Department of Pediatrics, University of Virginia School of Medicine, Charlottesville, VA 22903 USA

**Keywords:** Cystic fibrosis, CFTR, Kidney, High-efficiency modulator therapy, AKI, CKD, CFKD

## Abstract

Cystic fibrosis (CF) is a life-shortening multisystem disease resulting from mutations in the cystic fibrosis transmembrane conductance regulator (CFTR) gene, causing the most devastating phenotypes in the airway and pancreas. Significant advances in treatment for CF lung disease, including the expanded use of high-efficiency modulator therapies (HEMT) such as Trikafta, have dramatically increased both quality of life and life expectancy for people with CF (PwCF). With these advances, long-term extrapulmonary manifestations are more frequently recognized. Pseudo-Barter syndrome, acute kidney injury (AKI) induced by medications or dehydration, amyloidosis, nephrolithiasis, and IgA and diabetic nephropathies have been previously reported in PwCF. Newer data suggest that chronic kidney disease (CKD) is a new morbidity in the aging CF population, affecting 19% of people over age 55. CKD carries a high risk of premature death from cardiovascular complications. Studies suggest that CFTR dysfunction increases kidneys’ vulnerability to injury caused by the downstream effects of CF. Improving the mutant CFTR function by HEMT may help to tease apart the kidney responses resulting from extrinsic factors and those intrinsically related to the *CFTR* gene mutations. Additionally, given the novelty of HEMT approaches, the potential off-target effects of their long-term use are currently unknown. We review the evolving kidney complications in PwCF and propose the term CF-related kidney disease. We hope this review will increase awareness about the changing phenotype of kidney dysfunction in PwCF and help prevent morbidity related to this condition.

**Graphical abstract:**

**Figure:**
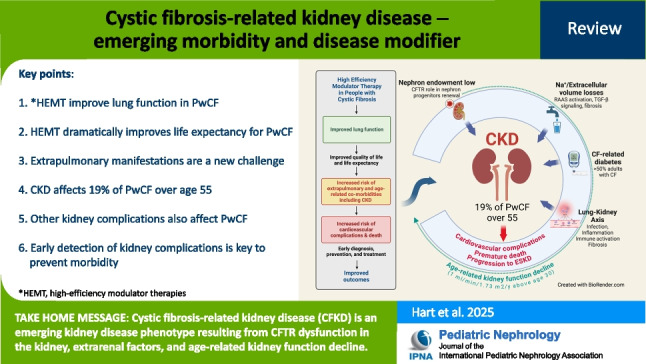
A higher resolution version of the Graphical abstract is available as Supplementary information

**Supplementary Information:**

The online version contains supplementary material available at 10.1007/s00467-025-06715-3.

## Introduction

Cystic fibrosis (CF) is a life-shortening multisystem autosomal recessive disease resulting from mutations in the cystic fibrosis transmembrane conductance regulator (CFTR) gene affecting nearly 40,000 people in the US and 160,000 individuals worldwide [1, 2]. CF affects the exocrine epithelial tissues and has the most devastating phenotype in the airway and pancreas. CFTR (ABCC7) protein is an ATP-binding cassette (ABC) transporter family member with phosphorylation-activated, ATP-gated anion channel function [3]. CFTR is expressed in most fluid-transporting epithelial tissues, spanning the apical plasma membrane of polarized cells, except in the sweat gland, where CFTR is expressed in both the clear cells’ apical and basolateral membrane domains. CFTR plays a significant role in secretory epithelia where it mediates Cl^−^ and HC03^−^ secretion with a 5:1 conductive ratio for Cl^−^ and HC0_3_^−^, respectively [4]. The luminal Cl^−^ secretion drives Na^+^ movement across the epithelium via a paracellular route, and the increased luminal salt concentration creates an osmotic driving force for water movement either passively via paracellular pathways or transcellularly through aquaporins, resulting in isotonic secretions. Under a normal electrochemical gradient, CFTR-mediated Cl^−^ transport accounts for most of the fluid secretion in most secretory tissues. CFTR-mediated HC0_3_^−^ secretion also contributes to transepithelial fluid secretion, acid/base regulation, mucus function, and host defensins [5, 6].

Since the first description of CF by Dr. Dorothy Anderson in 1938, many ground-breaking discoveries have led to the development of medications extending the life expectancy to adulthood for most people with CF (PwCF) [7, 8]. The cloning of the *CFTR* gene in 1989 allowed discoveries of the CF-causing mutations and CFTR protein structure and function, along with the identification of modifiers contributing to the high phenotypic variability of CF [9] CF Foundation (CFF) Therapeutics Development Network was launched in 1998 to develop CF-modifying therapies. The therapeutics, collectively known as CFTR modulators, target the underlying defect in CFTR protein, are administered enterally, and include two classes of medications, reviewed by Mall et al. [9]. Correctors improve the processing and allow CFTR protein to reach the cell surface, while potentiators activate the CFTR channel. The first potentiator, ivacaftor, was approved by the Food and Drug Administration (FDA) in 2012, and ultimately, the combined potentiator therapy with two correctors, Trikafta, in 2019.

The disease-causing *CFTR* gene mutations are classified into six groups according to their structural or functional consequences. The most prevalent mutation, in-frame deletion of three bases encoding a phenylalanine residue at position (F508del), likely originated in Western Europe during the Bronze Age [10]. This mutation, present in approximately 70% of PwCF, impairs the folding and maturation of CFTR protein, followed by its degradation rather than trafficking to the cell surface [11]. Thus, PwCF carrying F508del-CFTR or similar mutations benefit from both the corrector and potentiator activity of Trikafta. By contrast, the G551D substitution inactivates the CFTR channel without disrupting the protein trafficking. Thus, patients carrying G551D-CFTR benefit from the potentiator activity of ivacaftor. Several rare CFTR mutants do not respond to modulators and await more therapeutic discoveries. Yet for most PwCF who benefit from the improved quality of life and increased life expectancy, CF has become a chronic condition of adulthood with long-term extrapulmonary CF manifestations and age-related co-morbidities, including diabetes, liver and bone disease, hypertension, and cancer [12]. Since the number of PwCF afflicted by these co-morbidities is rising, CF care has been shifting from preventing mortality to preventing morbidity. One of the emerging challenges in PwCF is chronic kidney disease (CKD). Here, we discuss the evolving phenotype and risk factors of kidney dysfunction in PwCF.

### Kidney complications in PwCF

Acute kidney injury (AKI), amyloidosis, nephrolithiasis, IgA nephropathy (IgAN), and diabetic glomerulopathy are known complications in PwCF [13]. Between 1957 and 1983, systemic amyloid deposition was reported at autopsy in 33% (*n* = 11/33) of PwCF older than 15, but these patients died of other CF complications before developing clinically detectable amyloidosis [14]. Amyloid A (AA) amyloidosis and diabetic glomerulopathy were the predominant diagnoses in kidney biopsies performed in 13 of 512 adult PwCF (*n* = 3, each) between 1992 and 2008 [15]. AA amyloidosis affects 1–2 people per million person-years and has a poor prognosis, and PwCF are at risk due to chronic infection and inflammation [16]. By contrast, in a series of 21 native kidney biopsies published in 2024, the most frequent diagnoses were acute tubular injury (*n* = 10) and IgAN (*n* = 8), while diabetic glomerulopathy was diagnosed less frequently (*n* = 2), and there were no cases of amyloidosis [17]. Acute tubular injury was associated with the use of antibiotics. Better management of chronic infection and inflammation may be responsible for the decreased frequency of amyloidosis. It has been speculated that the IgAN in PwCF is associated with mucosal infections, inflammation, liver disease, or medications in PwCF [13, 17]. While this may be correct, no data compare the prevalence of the most common worldwide glomerular disease between PwCF and the general population [18]. Nevertheless, IgAN has become the most common glomerular disease in PwCF. Independent of the primary histologic diagnosis in this biopsy series, most patients also had chronic changes, including global glomerulosclerosis, interstitial fibrosis, and tubular atrophy, and many patients progressed to CKD or kidney failure [17]. The above observations indicate that kidney complications evolve parallel with the changing management and remain a significant extrapulmonary manifestation in the aging CF population.

Advancing age is an independent risk factor for CKD, with a median decrease in the estimated glomerular filtration rate (eGFR) by 1 ml/min/1.73 m^2^/year per year after age 30 [19, 20]. The age-adjusted prevalence estimates for CKD are about two-fold higher in PwCF compared to the general population [21]. The annual prevalence of moderate (stage 3) CKD in PwCF was 2% and doubled every 10-year increase in age, reaching 19% in people over 55 [21]. As such, CKD has been proposed as a new morbidity in aging PwCF [22]. PwCF have become increasingly aware of kidney disease in their community and coined the term CF-associated kidney disease (CFKD), reflecting the emerging challenge of the new CF co-morbidity [23]. In a study of 226 PwCF, 50% of males and 66% of females had abnormal eGFR, either decreased, suggesting loss of function, or increased, suggesting kidney hyperfiltration [24]. PwCF with CKD were generally older, diabetic, with longer medial duration of chronic lung infection, and with longer intravenous aminoglycoside use. Older age and higher levels of uric acid, triglycerides, and total and LDL cholesterol correlated with decreased eGFR. CF patients with lung transplants had significantly lower eGFR than those without transplants, while their forced expiratory volume in 1 s (FEV1) did not differ. These results emphasize the kidney vulnerability in PwCF, potentially exacerbated by decreased kidney reserve.

CF-related diabetes (CFRD) affects more than 50% of adult PwCF and is the most common non-pulmonary co-morbidity [25]. The microvascular complications in patients with CFRD were like those seen in people with diabetes mellitus type 1, but PwCF had a significantly higher prevalence of albuminuria (21% vs. 4%, *p* = 0.003), suggesting other CF-related factors affecting kidney function [26]. Among 181 Danish PwCF, the CKD prevalence was 2.7%, increasing to 11% after the inclusion of lung transplant patients [27]. While exposure to aminoglycoside antibiotics is a well-known risk factor for AKI, not all studies have confirmed the correlation between eGFR loss and cumulative life-long exposure to aminoglycosides [28, 29]. Although the frequent use of aminoglycoside antibiotics and episodes of dehydration have contributed to CKD in some PwCF, a significant number of kidney failure cases have no identified cause [30]. Early CKD detection is critical to reduce the risk of premature death caused primarily by heart disease [31]. However, CKD is frequently diagnosed during advanced stages because clinical symptoms emerge slowly and silently [32–35]. This has been clearly demonstrated in PwCF with chronic lung disease who had evidence of pre-clinical kidney injury despite normal eGFR [36]. The rate of cardiovascular disease in PwCF is reported to be between 10 and 30% [37]. It varies among individuals, and studies and may be underreported. It is unknown how CFKD modulates the cardiovascular risk in PwCF.

Other manifestations of kidney disease in PwCF include hypercalciuria, nephrocalcinosis, and nephrolithiasis. Nephrocalcinosis and hypercalcuria are prevalent in young children with CF [38, 39]. Microscopic nephrocalcinosis was observed in 35 of the 38 kidney specimens (92%) and hypercalciuria (> 0.182 mg/mg of creatinine) in 5 of the 14 patients (36%) [38]. The timing of nephrocalcinosis detection near birth in these patients led to the hypothesis that kidney Ca^2+^ deposition resulted from the consequences of CFTR dysfunction rather than longstanding pulmonary dysfunction, chronic infection, therapeutic agents, or disease progression [38]. However, not all studies confirmed such a high prevalence of nephrocalcinosis in neonates with CF [40]. Hypercalcuria and nephrocalcinosis frequently result from impaired function of transporters in the kidney tubules dependent on CFTR function, including V-ATPase and pendrin (Cl^−^/HCO3^−^ exchanger) [41, 42]. Other contributing factors are present as well, including hypocitraturia [43]. Recurrent calcium oxalate stones are three times more common in PwCF than in the general population and affect all ages [43–45]. Despite the alarming statistics, the mechanisms of these manifestations in PwCF are poorly understood. Kidney stones are a significant cause of morbidity. The kidney complications in PwCF are summarized in Table 1.

**Table 1:** Summary of kidney complications in PwCF

Kidney complications	Mechanisms
Pseudo-Barter syndrome (hyponatremia, hypochloremia, hypokalemia, metabolic alkalosis)	Secondary hyperaldosteronism from excessive Na^+^ and fluid loss via sweat Impaired function of pendrin caused by CFTR dysfunction in ICB in CD
AKI	Prerenal azotemia Nephrotoxic drug exposure (aminoglycosides, NSAIDs)
AA amyloidosis	Glomerular AA amyloid deposition
IgA nephropathy	Mucosal infection, inflammation, liver disease, medications
Diabetic glomerulopathy	CFRD
Tubulointerstitial nephritis	Medications
CKD (< eGFR, albuminuria) (19% of PwCF > 55 y) Histological features: -Tubulointerstitial fibrosis -Tubular atrophy -Glomerulosclerosis	Decreased nephron endowment Chronic or recurrent Na^+^ loss via sweat CF lung disease and pulmonary exacerbations Inflammation and neutrophil-mediated tissue injury CFRD TGF-β pathway activation Age-related kidney function loss Genetic risks factors
LMW (non-glomerular) proteinuria	Loss of CFTR-mediated endosomal acidification in PT cells leading to impaired absorption of LMW proteins Tubulointerstitial fibrosis
Vitamin D deficiency/osteopenia	Urinary loss of vitamin D–binding protein and urinary loss of vitamin D
Iron deficiency anemia	Urinary loss of vitamin transferrin
Hypercalcuria/nephrolithiasis/nephrocalcinosis	Impaired function of pendrin caused by CFTR dysfunction in ICB of CD Hypocitraturia

*AA*, amyloid A; *ANG*, Angiotensin; *CFRD*, CF-related diabetes; *CFKD*, CF-related kidney disease; *ICB*, B-type intercalated cells; *CD*, collecting duct; *NSAIDs*, non-steroidal anti-inflammatory drugs; *IgA*, immunoglobulin A; *LMW*, low molecular weight; *PT*, proximal tubule; *RAAS*, renin–angiotensin–aldosterone; *TGF-β*, transforming growth factor-β

### Risks of kidney complications in PwCF

#### Altered nephron endowment and decreased kidney reserve

CFTR mRNA and protein are expressed in most nephron segments except the glomeruli in humans and animal models [46–52]. The earliest appearance of CFTR protein during human kidney development was noted in the apical region of the branching ureteric bud epithelial cells, coinciding with the immunoreactivity for the AQP2 water channel (12-week gestation) [46]. The expression of CFTR in the ureteric bud correlated with its expression in other organs undergoing branching morphogenesis, including the lung, pancreas, bile duct, and salivary gland [53, 54]. By 15 weeks, CFTR was also diffusely expressed throughout the cytoplasm of proximal tubules and loops of Henle, correlating with the morphological maturation of these tubular structures originating from the metanephric mesenchyme marked by the appearance of AQP1 water channel immunostaining in the proximal tubule. From 15 to 24 weeks of gestation (the end of the 2nd trimester), this staining pattern remained constant and included immunoreactivity of the transitional epithelium of the pelvicalyceal system. Based on the above findings, CFTR was proposed as an early marker of tubular differentiation, like AQP1 and AQP2 [55]. Glomerular filtration starts between 9 and 12 weeks of gestation and results in tubular function by the end of the 2nd trimester. It is unknown if CFTR is functionally active at this stage. However, a CFTR-like Cl^−^ channel activity was seen in the 2nd trimester of human epididymal cells [56]. Thus, it was hypothesized but not experimentally validated that CFTR could play a role in fluid secretion and lumen formation of the expanding ureteric bud and kidney tubule maturation [46]. In addition to the full-length CFTR, a truncated CFTR splice variant is expressed in a specific pattern, starting from week 13 of gestation and reaching the postnatal level at the end of the 2nd trimester when its abundance increased tenfold over the full-length CFTR and was predominantly localized to the kidney medulla [46, 51]. These results show the complex regulation of CFTR during nephrogenesis and suggest the respective roles of the full-length and the splice variant CFTR proteins in the human kidney at the plasma membrane and in intracellular organelles [57]. After birth, the kidney tubules demonstrate primarily a CFTR-independent absorptive phenotype that remains intact in PwCF who adapt to intravascular volume contraction from excessive Na^+^ losses in sweat.

It was previously concluded that CFTR does not play a direct role in nephrogenesis because CFTR protein was not found in the comma- and S-shaped bodies in the nephrogenic zone [46]. However, a more recent study in a murine model showed that CFTR may play a role in regulating the nephron number or endowment [58]. Nephron progenitors give rise to multiple nephron segments, including podocytes, proximal tubules, distal tubules, and loops of Henle. Strictly orchestrated signaling events regulate the differentiation of nephron progenitors into these kidney structures [59]. Nephron progenitors also undergo self-renewal to prevent premature depletion before the cessation of nephrogenesis. In a mouse model, the ablation of nephron progenitors in utero resulted in reduced nephron endowment [60, 61]. Decreased kidney reserve, resulting from the formation of fewer or abnormal nephrons during development, is a significant risk factor for increased kidney vulnerability to injury and CKD [62, 63]. Micro-(mi)RNAs regulate nephron progenitors [64]. Conditional deletion of the miR-17 ~ 92 cluster in nephron progenitors resulted in kidney hypodysplasia, proteinuria, and CKD in a mouse model [65]. CFTR was found to be the most differentially repressed gene target of miR-17–92 [58]. Pharmacological inhibition of CFTR activity in nephron progenitor cultures impaired cellular proliferation, and miR-17 ~ 92-null progenitors exhibited disrupted cellular proliferation that resulted in low nephron endowment. These data suggest that CFTR dysfunction alters nephron endowment, contributing to increased incidence of CKD in the aging CF population.

#### Impaired endocytic uptake of low molecular weight proteins in kidney proximal tubules

**Fig. 1:**
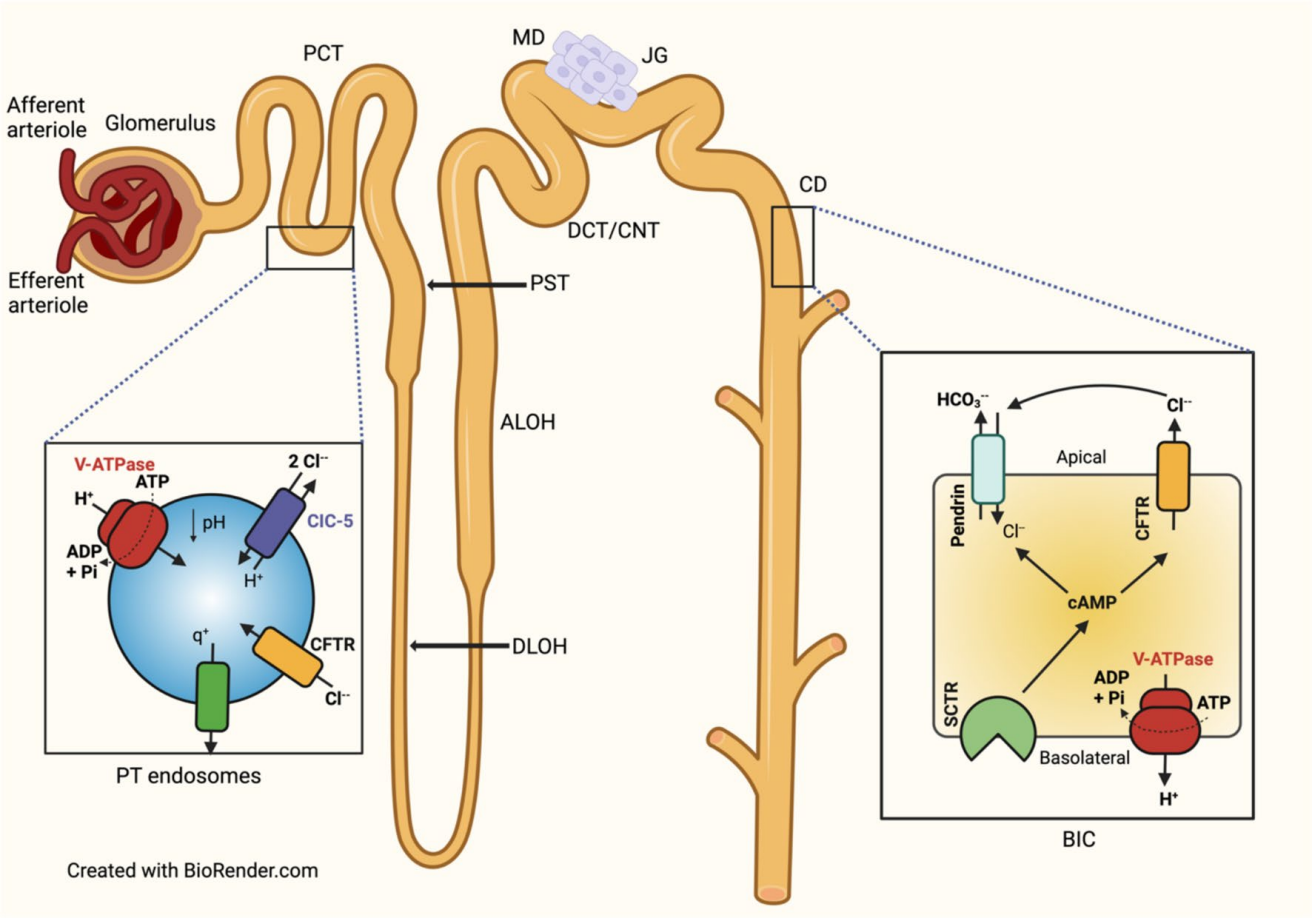
Proposed CFTR role in endosomal acidification in the proximal tubule (**A**) and HCO_3_^−^ secretion in the collecting duct (**B**) to proximal tubules, ATP-driven transport of cytosolic H^+^ through the vacuolar H^+^-ATPase (V-ATPase) in the proximal tubules. The endosomal chloride channel (CIC)−5 is a CI^−^/H^+^ exchanger (2 Cl^−^:1 H^+^) facilitating endosomal acidification. CFTR enriched in endosomal membranes and the cation leakage (q^+^) could participate in the electrical shunt necessary for sustained vesicular acidification (modified from Devuyst et al.) [71].In the collecting duct and connecting tubules, B-intercalated cells (BIC) secrete HCO_3_^−^. BIC express V-ATPase at the basolateral membrane. The rise of intracellular cAMP concentration following stimulation of the basolateral secretin receptor (SCTR) stimulates CFTR and pendrin activity and/or localization in the apical membrane and activates HCO_3_^−^ secretion. HCO_3_^−^ secretion occurs via pendrin, which also drives Cl^−^ reabsorption. A functional CFTR is required for pendrin function. Whether CFTR directly stimulates pendrin or simply provides a pathway for Cl^−^ recycling remains unknown (modified from Tamma et al.) [72]. Nephron segments: PCT, proximal convoluted tubule; PST, proximal straight tubule; DLOH, descending limb of Henle’s loop; ALOH, ascending limb of Henle’s loop; MD, *macula densa*; DCT/CNT, distal convoluted tubule/connecting duct; JG, juxtaglomerular apparatus; CD, collecting duct

CFTR, distributed at the apical endosomes of PT cells, is thought to play a role in the endocytic uptake of low molecular weight (LMW) proteins (Fig. 1) [47]. LMW proteinuria has been noted in PwCF [36, 49]. CFTR knockout (KO; Cftrtm1Cam) mice showed a significant increase in the urinary excretion of the LMW Clara cell protein (16 kDa), defective uptake of ^125^I-β2-microglobulin, and abnormal shedding of cubulin in the urine, associated with urinary loss of ligands, such as transferrin [49]. The urinary loss could contribute to lower circulating transferrin levels and iron deficiency observed in PwCF [66]. Similarly, the urinary loss of vitamin D binding protein could explain its decreased serum levels and impaired vitamin D reabsorption by the megalin/cubulin receptor in the PT and vitamin D deficiency observed in pediatric and adult PwCF [67–69]. Vitamin D deficiency is a common finding in PwCF despite supplementation. It is associated with a high prevalence of osteopenia, osteoporosis, and increased morbidity, including fractures, kyphosis, and worsening pulmonary status [70].

#### Impaired HC03^−^ secretion in the collecting duct of B-type intercalated cells

The kidney cortical collecting duct and connecting tubule intercalated cells (IC) are essential regulators of acid–base homeostasis. This function depends on the differential cell surface expression of vacuolar-type H^+^-ATPase (V-ATPase) on the luminal surface in A-type IC (AIC) and the serosal surface in B-type IC (BIC). The apical Cl^−^/HC03^−^ exchanger pendrin (Slc26A4, PDS) located in BICs regulates acid–base balance by excreting HC03^−^ and reabsorbing Cl^−^ followed by Cl^−^ secretion by CFTR (Fig. 1) [42, 73]. CFTR dysfunction impairs pendrin-mediated HC03^−^ secretion and predisposes PwCF and CFTR knockout (KO) mice to metabolic alkalosis [74, 75]. In control mice, acute oral HC03^−^ loading induced a dose-dependent metabolic alkalosis followed by fast urinary excretion of HC03^−^ without affecting ventilation. By contrast, CFTR or pendrin KO mice could not rapidly excrete excess HC03^−^ and developed transient hypoventilation when subjected to the same HC03^−^ load [76]. Thus, it has been hypothesized that CFTR dysfunction may reduce the respiratory drive and cause hypoventilation in PwCF during episodes of metabolic alkalosis, leading to more severe lung dysfunction and hypercapnic respiratory failure. CFTR dysfunction leads to chronic metabolic alkalosis after prolonged oral loading with HC03^−^ [77, 78]. Thus, metabolic alkalosis observed in PwCF may be a result of the absence of CFTR-mediated Cl^−^ recycling in this nephron segment [75, 79]. Impaired Cl^−^/HC03^−^ exchange by pendrin in PwCF may also lead to renal Ca^2+^ wasting and hypercalciuria, as discussed earlier.

#### Intravascular volume contracture

Loss of CFTR function leads to chronic or recurrent excessive Na^+^ loss via sweat and may increase the risk for TGF-β-mediated kidney fibrosis in PwCF. Na^+^ loss predisposes to intravascular volume depletion and activates the renin–angiotensin–aldosterone system (RAAS). Stimulation of plasma renin activity and aldosterone concentration supports increased kidney Na^+^ absorption, volume preservation, and maintenance of blood pressure [80]. When combined with nephrotoxic medications, repeated episodes of fluid and Na^+^ losses and intravascular volume depletion can cause repeated episodes of kidney injury and eventually lead to CKD. Renin also acts as an angiotensin-independent pro-fibrotic mediator, while angiotensin (ANG) II stimulates TGF-β expression and upregulates receptors for TGF-β in the kidney by various mechanisms. ANG II can directly activate the TGF-β pathway through Smads and without altering latent TGF-β protein levels. Other components of the RAAS, including ANG III and aldosterone, also activate the TGF-β system. TGF-β is the most essential profibrotic pathway in the kidney and a significant driver of CKD progression. PwCF have elevated plasma renin activity and aldosterone concentrations [80, 81]. Gene polymorphisms for both the receptor for the *ANG II* (*AGTR2*) and *TGF-β* genes are modifiers of CF disease progression [82, 83]. The presence of tubulointerstitial fibrosis, which affects the processing of LMW proteins by the kidney tubules, would explain the finding that PwCF have higher proportions of LMW proteins in urine [48]. While previous studies have suggested that CFTR dysfunction leads to TGF-β mediated kidney fibrosis [24, 84], the intravascular volume depletion and Na^+^ loss may represent one of the upstream drivers for TGF-β signaling in PwCF.

#### Nephrotoxic drug exposure

The role of nephrotoxic drug exposure in the pathogenesis of CKD in PwCF remains unclear. Despite rigorous therapeutic monitoring, the incidence of nephrotoxicity and AKI after aminoglycoside use in the general population is 10–25% [85]. The incidence is similar in PwCF [86]. While some studies support the conclusion that high doses of aminoglycosides trigger AKI in PwCF [28, 87], contrary evidence must also be considered [88]. One study showed the presence of CKD in a cohort of PwCF was not related to prolonged aminoglycoside use [21, 86]. In other studies, recurrent aminoglycoside exposure contributed to CKD in adult PwCF but not in children with CF [89, 90]. Studies examining the association between aminoglycoside use and kidney injury during CF pulmonary exacerbation often do not account for the inflammatory events driving the exacerbation [29]. Although AKI and CKD prevalence is markedly increased in PwCF, kidney disease presentation is heterogeneous both between patients and within the same patient across separate admissions. The question of why some aminoglycoside courses lead to AKI despite dose adjustment or maintaining the hydration status, but others do not despite recurrent exposure lies among many other unknowns. Aminoglycoside nephrotoxicity has a complex mechanism involving tubular damage (apoptosis or necrosis) as well as oxidative stress, inflammation (neutrophil infiltration), and glomerular injury, leading to vasoconstriction and reduced glomerular filtration. The above data indicate that aminoglycoside nephrotoxicity alone does not fully explain the vulnerability of PwCF to CKD. Since pulmonary exacerbations are often treated with nephrotoxic doses of aminoglycosides, the contribution of treatment vs. inflammatory events caused by lung infection to AKI and, ultimately, CKD in PwCF is unknown.

#### CF lung disease and the lung-kidney axis

The role of the lung-kidney axis has been well-documented in different clinical scenarios [91, 92]. However, the effects of CF lung disease on kidney function are incompletely understood. Our group recently described several novel associations between lung disease and kidney injury in 48 PwCF, of whom 60% were treated with HEMT [36]. We demonstrated a correlation between *P. aeruginosa* lung infection and urine kidney injury marker (KIM)−1. High urinary KIM-1 levels and increased prevalence of neutrophils among urine immune cells correlated with decreased lung function. These data indicate that inflammation and neutrophilic infiltration, linked to worse lung function and pulmonary exacerbations, may contribute to kidney injury even in patients treated with HEMT. In this study, the kidney injury markers did not correlate independently with aminoglycoside (inhaled tobramycin) therapy or CFRD, emphasizing that the lung-kidney axis was independent of the potential confounders. CF neutrophils often cannot eradicate lung infection due to decreased phagocytic capacity [93]. Instead, the increased oxidative burst and release of proteases are thought to contribute to neutrophil-mediated tissue injury, including the propagation of fibrotic processes [94, 95]. Besides its role in immune cell activation, CFTR may play a role in metabolic pathways and endothelial cell function that are more linked to kidney fibrosis by dysregulating Wnt/β-catenin and TGF-β signaling [84]. Oxidative stress and fibrosis or coagulopathy from acid–base imbalance may contribute to the functional decline across the lung-kidney axis [91]. The above data demonstrate that kidneys in PwCF are vulnerable to damage during pulmonary exacerbations or chronic lung disease.

#### Genetic factors

The severity of CF lung disease can be modified by genetic polymorphisms. It can be predicted that modifiers associated with worse lung function may also negatively impact kidney function in PwCF. No genome-wide association studies (GWAS) have been published to identify genetic modifiers crucial for the development and progression of CKD in PwCF. Still, separate studies have identified potential genetic modifiers common to both diseases. Although a full review of this topic is beyond the scope of this manuscript, it is worth mentioning that TGF-β is one of the most established negative genetic modifiers in CF and a major profibrotic mediator in kidney disease. Its genetic variations are also associated with susceptibility to IgAN and CKD [96, 97]. Genetic polymorphisms are associated with diabetic nephropathy, but the genetic risk of chronic kidney dysfunction in CFR is unknown.

### Effects of HEMT on kidney function

The effects of HEMT on kidney function have not yet been studied systematically. Components of the FDA-approved Trikafta triple therapy (elexacaftor, tezacaftor, and ivacaftor) are substrates for cytochrome CYP3A metabolized by the liver and excreted in feces. Their kidney excretion is minimal. A prospective single-center observational study evaluated the effects of triple therapy on kidney function in 19 adults with CF over seven months [98]. There was no significant change in eGFR or urine total protein, albumin, and β2-microglobulin excretion. Serum aldosterone level and aldosterone/renin ratio decreased significantly, likely reflecting fluid loss reduction through sweat. There was no change in metabolic risk factors for nephrolithiasis. Interestingly, vitamin D levels increased while dietary calcium intake decreased. Decreased urinary loss of vitamin D–binding protein and improved vitamin D reabsorption by the megalin/cubulin receptor in the PT could explain improved vitamin D levels [67–69]. There was no significant change in systolic and diastolic blood pressure. Although this study provides the first report of the effects of HEMT on kidney function in PwCF, the patient selection, small subject number, and short follow-up limit the interpretation of the results. Future studies will also need to determine if potentiating CFTR function by HEMT will restore the CFTR balance during kidney development.

## Closing remarks

Life expectancy has improved for PwCF. CKD is a new morbidity, affecting 19% of PwCF over age 55. The risks of CKD and other kidney complications involve CFTR dysfunction in the kidney, CF extrarenal factors, and age-related kidney function decline. We propose broadly using CFKD to define CKD and other chronic kidney complications in PwCF. Recent data suggest that standard clinical tests may delay the detection of CFKD. In the general population, CKD is a significant risk for cardiovascular disease and premature death. We propose that early recognition and prevention of CFKD are essential for preventing cardiovascular consequences and improving the quality of life in PwCF. Nephrologists and urologists have a new opportunity to help reduce CF morbidity. More studies are needed to face the challenges of continuing to improve the lives of PwCF.

## Supplementary Information

Below is the link to the electronic supplementary material.

Graphical abstract (PPTX 456 MB)
