# Supplementary material for: Cystic fibrosis–related kidney disease—emerging morbidity and disease modifier
Source: Pediatr Nephrol. 2025 Mar 17;40(12):3605–14. doi: 10.1007/s00467-025-06715-3 (PMC12549735; doi:10.1007/s00467-025-06715-3)
Supplement: Supplementary file 1 — Graphical abstract (PPTX 456 MB) [file 467_2025_6715_MOESM1_ESM.pptx]

## Slide 1
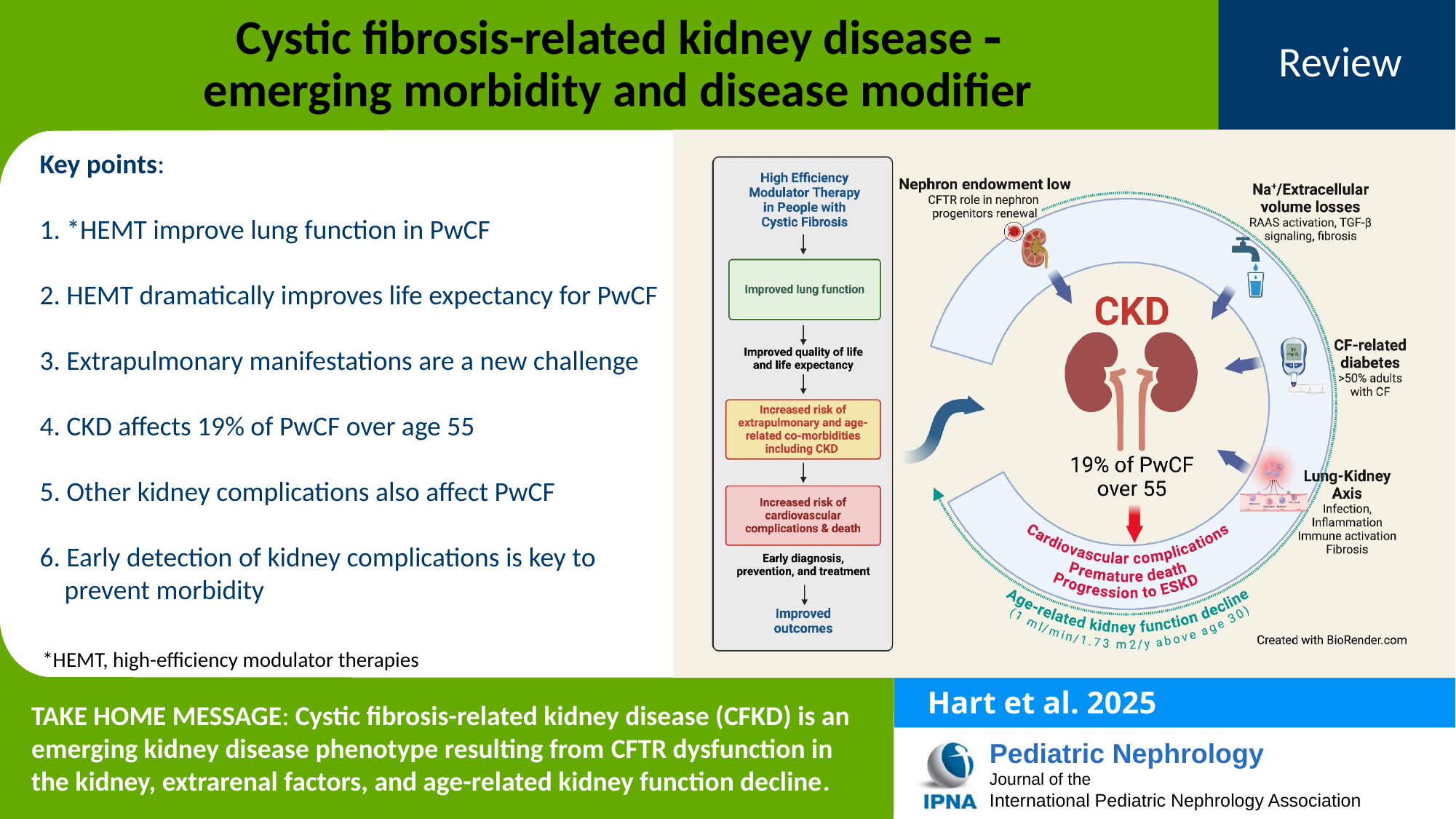

Cystic fibrosis-related kidney disease −
emerging morbidity and disease modifier
Key points:
1. *HEMT improve lung function in PwCF
2. HEMT dramatically improves life expectancy for PwCF
3. Extrapulmonary manifestations are a new challenge
4. CKD affects 19% of PwCF over age 55
5. Other kidney complications also affect PwCF
6. Early detection of kidney complications is key to
 prevent morbidity
*HEMT, high-efficiency modulator therapies
Hart et al. 2025
TAKE HOME MESSAGE: Cystic fibrosis-related kidney disease (CFKD) is an emerging kidney disease phenotype resulting from CFTR dysfunction in the kidney, extrarenal factors, and age-related kidney function decline.
